# Use of Procalcitonin in Patients on Chronic Hemodialysis: Procalcitonin Is Not Related with Increased Serum Calcitonin

**DOI:** 10.5402/2012/431859

**Published:** 2012-05-20

**Authors:** Ken-Ichi Mori, Mitsuru Noguchi, Yasuhiro Sumino, Fuminori Sato, Hiromitsu Mimata

**Affiliations:** ^1^Department of Urology, Faculty of Medicine, Oita University, Idaigaoka 1-1, Hasama-cho, Yufu City 879-5593, Oita, Japan; ^2^Department of Urology, Saga University Faculty of Medicine, Saga 849-8501, Japan

## Abstract

*Objectives*. To investigate whether procalcitonin (PCT) could be useful for detecting bacterial infections in patients on hemodialysis (HD) and with increased calcitonin (CT). *Methods*. This prospective study included 42 males and 34 females on HD. The infection group consisted of 15 patients with proven bacterial infections; the other 61 patients were designated as the noninfection group. Serum C-reactive protein (CRP), interleukin (IL)-6, white blood cell (WBC) count, immature and total neutrophil (I/T) ratio, and CT were measured at the beginning of HD, and serum PCT levels at the beginning of HD and after HD. *Results*. The mean CT level in the both groups was apparently higher than that of nonchronic kidney disease. Significantly higher values between the infection and noninfection groups were seen for CRP, IL-6, WBC, I/T ratio, PCT, and CT. The PCT value of the area under the receiver operating characteristic curve was 0.921, which was significantly higher than the values for CRP (0.853; *P* < 0.01), IL-6 (0.739; *P* < 0.01), WBC (0.692; *P* < 0.01), and I/T ratio (0.584; *P* < 0.01). *Conclusions*. PCT was useful marker of bacterial infection in patients on HD and with increased CT. PCT levels should be determined before HD.

## 1. Introduction

Procalcitonin (PCT) was first described in 1993 as a diagnostic marker of infection [1]. It is a polypeptide of 116 amino acids and has a molecular weight of 13 kDa. The clinical significance of serum PCT in discriminating between bacterial infections and nonbacterial infections, such as systemic inflammatory response syndrome (SIRS), has been compared with that of other markers including endotoxin, *β*-D-glucan, interleukin (IL)-6, C-reactive protein (CRP), and white blood cell (WBC) count [2–6]. Serum PCT levels in patients with systemic or localized bacterial infections were significantly higher than those in patients with nonbacterial infections or noninfectious diseases [7]. Furthermore, PCT-guided antibiotic therapy for respiratory tract infections has been shown to significantly decrease antibiotic exposure with subsequent reduction in antibiotic-associated side effects and antibiotic resistance [8]. PCT is also an effective tool for diagnosis and risk stratification of upper urinary tract infections in children [9, 10]. Based on these findings, PCT is now identified as an important marker of bacterial infections.

PCT is the precursor molecule of human calcitonin (CT) and is synthesized by thyroid C cells. Increased serum CT levels have been observed in patients with chronic kidney disease due to increased secretion of CT by thyroid C cells, rather than decreased glomerular clearance [11, 12]. Under conditions involving increased CT secretion, PCT may not function as a primary marker for detection of systemic and local bacterial infections in patients with chronic kidney disease.

 The use of PCT as a bacterial diagnostic marker is controversial in patients on hemodialysis (HD) [13–15]. Infections account for considerable morbidity and mortality in patients on HD [16]; hence, early diagnosis of bacterial infections is important to make a prognostic assessment of its severity. If PCT can be used as a primary marker for bacterial infections in patients on HD, these infections can be diagnosed and treated early. Therefore, we investigated whether PCT could function as a primary diagnostic marker of bacterial infections in patients on HD.

We first investigated whether PCT was useful in the diagnosis of bacterial infections in patients with increased serum CT levels and on HD. Furthermore, we investigated whether serum PCT levels were influenced by HD and determined the cut-off level of PCT in patients on HD.

## 2. Methods

### 2.1. Patient Population

The study was performed in accordance with the guidelines of the Declaration of Helsinki and was approved by the Izuhara Hospital Institutional Review Board in the Nagasaki Prefecture. Written informed consent was obtained from all patients. The study included 42 male and 34 female patients (median age 69 years, range 28–86) who underwent HD between April 2008 and March 2009. The patients were divided into 2 groups on the basis of a diagnostic work up. The first group (Infection group) included patients with confirmed systemic or localized (pneumonia or arteriovenous graft infection) bacterial infections. With or without positive sputum culture results, patients with pneumonia were defined as those with clinical signs and symptoms suggestive of lower respiratory infections, those showing consolidation on a plain chest radiograph. Bacterial infection involving an arteriovenous graft was diagnosed as a subcutaneous abscess associated with tenderness with positive bacterial culture results. Systemic bacterial infection was diagnosed in patients with high fever and positive blood culture results. The second group (noninfection group) included patients without suspicion of having an infection, and we selected randomly the control group consist of routine HD patients.

### 2.2. Laboratory Examinations

Serum CT, CRP, IL-6, WBC count, and immature and total neutrophil (I/T) ratio were measured at the beginning of HD. Serum PCT levels were measured at the beginning and 4 h after HD. HD was performed using high-flux membranes such as ethylene vinyl alcohol copolymer and polymethyl methacrylate membranes.

#### 2.2.1. PCT

Serum PCT levels in patients was determined by an electrochemiluminescence immunoassay (Procalcitonin Kit, Roche Diagnostics GmbH, Mannheim, Germany) that had a lower detection limit of 0.02 ng/mL. Levels below this detection limit were classified as “zero.” The intra-assay coefficient of variation was <10%. Two days were needed for a PCT measurement.

#### 2.2.2. CRP

CRP was measured by a latex agglutination method (C-Reactive Protein Kit, A&T Corporation, Kanagawa, Japan) that had a lower detection limit of 0.02 mg/L and an intra-assay coefficient of variation was <10%.

#### 2.2.3. IL-6

IL-6 was measured by a chemiluminescent enzyme immunoassay (Human IL-6 CLEIA, Fujirebio Inc., Tokyo, Japan) that had a lower detection limit of 0.2 pg/mL.

#### 2.2.4. WBC Count and I/T Ratio

WBC count and I/T ratio were measured using a multiparameter automated hematology analyzer designed for *in vitro* diagnostic use to count and characterize blood cells (CELL-DYN Sapphire, Abbott Laboratories, Abbott Park, IL, USA).

#### 2.2.5. CT

CT was measured by a radioimmunoassay (Calcitonin Kit, Mitsubishi Chemical Medicine Corporation, Tokyo, Japan) that had a lower detection limit of 12.5 pg/mL and an intra-assay coefficient of variation was <10%.

### 2.3. Statistical Analysis

 The Mann-Whitney *U* test was used to compare the groups, while receiver operating characteristic (ROC) analysis was performed to evaluate the ability of PCT to discriminate between the 2 groups.

PCT levels at the beginning and after HD were compared using the Wilcoxon matched-pairs test. *P* < 0.05 was considered statistically significant. The data were expressed as mean ± standard deviation.

## 3. Results

**Figure 1:**
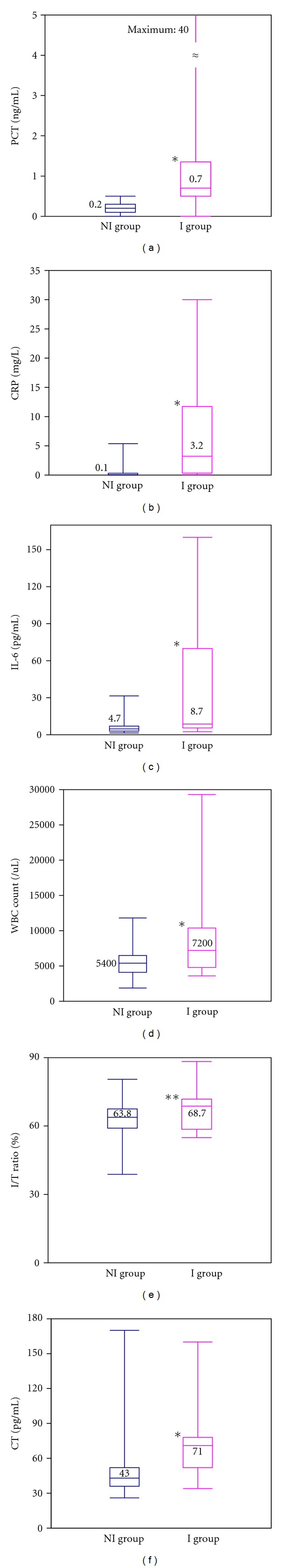
Comparison of box plots of clinical parameters before hemodialysis between the infection and noninfection groups. (1) PCT levels (*P < 0.01), (2) CRP levels (*P < 0.01), (3) IL-6 (*P < 0.01) levels, (4) WBC count (*P < 0.01), (5) I/T ratio (**P = 0.05), and (6) CT levels (*P < 0.01) were significantly higher in the Infection group than those in the Noninfection group. PCT: procalcitonin; CT: calcitonin; CRP: C-reactive protein; IL-6: interleukin-6; WBC: white blood cells; I/T ratio: immature and total neutrophil ratio; I: infection; NI: noninfection.

**Figure 2:**
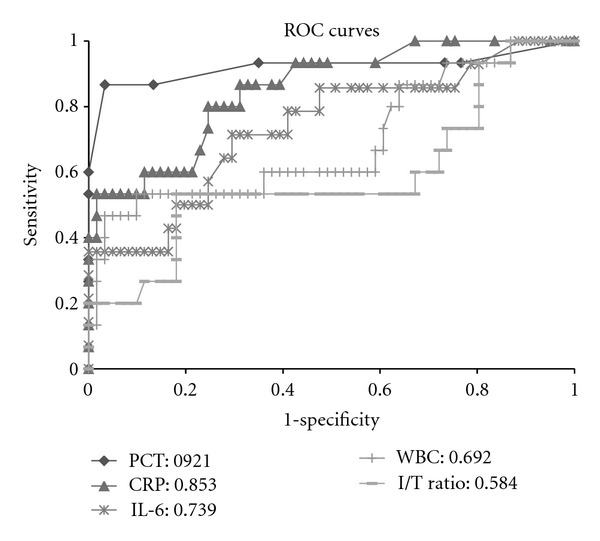
Receiver operating characteristic curves for PCT, CRP, IL-6, WBC, and I/T ratio. The area under the curve for PCT (0.921) was significantly higher than those for CRP (0.853; *P* < 0.05), IL-6 (0.739; *P* < 0.01), WBC (0.692; *P* < 0.01), and I/T ratio (0.584; *P* = 0.05). PCT: procalcitonin; CRP: C-reactive protein; IL-6: interleukin-6; WBC: white blood cell count; I/T ratio: immature and total neutrophil ratio.

The diagnostic work up identified 15 patients (10 males and 5 females, mean age 65.1 ± 14.9 years) with confirmed infections. Fourteen of them had localized bacterial infections and negative blood culture results. The localized infections were as follows: 13 patients had lower respiratory tract infections and 1 had bacterial infection involving an arteriovenous graft. One patient had systemic bacterial infection with positive blood culture results, and the patients had no identifiable source of infection. The remaining 61 patients selected randomly from routine HD patients (32 males and 29 females, mean age 65.7 ± 12.6 years), without suspicion of having an infection, were designated as the noninfection group. Significant differences in age and gender between the 2 groups were absent (Table 1). However, the infection group had significantly higher PCT (*P* < 0.01), CRP (*P* < 0.01), and IL-6 (*P* < 0.01) levels, WBC counts (*P* < 0.01), and I/T ratio (*P* = 0.05) than the noninfection group (Figure 1). The mean CT level in patients of both groups was apparently higher than that in patients with nonchronic kidney disease [17]. Furthermore, the mean CT level in the infection group was significantly higher than that in the noninfection group (*P* < 0.01) (Figure 1). A significant correlation was absent between PCT and CT levels in the Infection group but present in the Noninfection group (Table 2). In order to evaluate the various tests for discriminating between the 2 groups, area under the ROC curve (AUC) was calculated for each biomarker (Figure 2). AUC for PCT was 0.921, which was significantly higher than that for CRP (0.853; *P* < 0.01), IL-6 (0.739; *P* < 0.01), WBC (0.692; *P* < 0.01), and I/T ratio (0.584; *P* = 0.05). Using 0.5 ng/mL as the designated cut-off PCT level, the sensitivity and specificity were found to be 86.7% and 96.7%, respectively.

**Figure 3:**
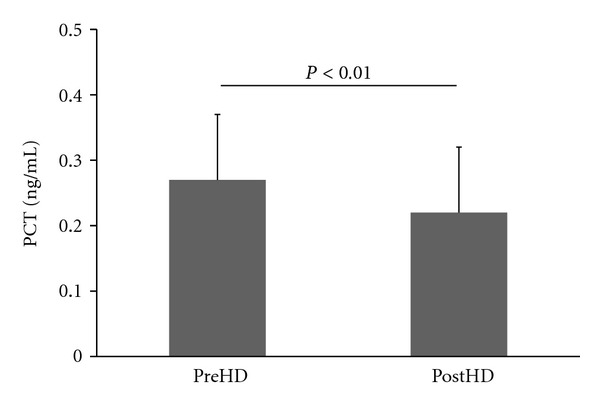
PCT levels before and after hemodialysis (HD). PCT levels decreased significantly after HD. The pre- and postHD measurements were compared using the Wilcoxon matched-pairs test. PCT: procalcitonin; preHD: before performing hemodialysis; postHD: after performing hemodialysis for 4 h.

HD was performed using high-flux membranes in all patients. PCT levels before and after HD were measured in 65 patients. The mean PCT level before HD was 0.27 ng/mL and that after HD was 0.22 ng/mL.  Figure 3 shows that PCT levels decreased significantly after HD (*P* < 0.01).

## 4. Discussion

Many investigators have reported that PCT is the most sensitive marker of bacterial infection [2–7]. Furthermore, Schuetz et al. reported that PCT-guided antibiotic therapy for respiratory tract infections can significantly reduce antibiotic exposure [8]. Recently, PCT was used not only as a marker of systemic bacterial infection but also as a marker of local bacterial infection. However, Dahaba et al. proposed that CRP, which is not affected by HD, may be a useful marker of sepsis in HD patients who have reduced PCT levels because of HD [15]. The use of PCT as a diagnostic marker of bacterial infections has been controversial in patients on HD. Bacterial infections are a major cause of mortality in patients on HD as these patients are often clinically compromised [16]. Therefore, it is important to diagnose bacterial infection at an early stage to improve prognosis.

Increased CT synthesis in thyroid C cells has been observed in patients on HD. Therefore, since PCT is the precursor molecule of CT, it is believed that PCT synthesis would also be increased. However, the relationship between PCT and CT in patients on HD has not been reported. Because of increased CT synthesis in thyroid C cells in patients on HD, we believe that PCT cut-off levels for indicating bacterial infection in these patients may be higher than those in patients without chronic kidney disease.

In this study, mean CT level in patients of both groups was higher than that in patients with nonchronic kidney disease [17]. However, we demonstrated that PCT was the most useful marker for differentiating bacterial infections from nonbacterial infections including those in patients on HD. The cut-off level for PCT was approximately 0.5 ng/mL, a value similar to that reported in a previous study on patients without chronic kidney disease [18, 19]. Serum PCT levels may increase under many conditions associated with SIRS, even in the absence of a thyroid gland [20]. Furthermore, PCT is synthesized in the small intestine, lungs, and liver in patients with bacterial infections [21, 22]. In the noninfection group, PCT was synthesized in the thyroid gland alone, thus, explaining the significant correlation between PCT and CT levels. However, it can be synthesized in the thyroid gland as well as in other organ such as lung in response to bacterial infections. PCT synthesized in lung may not be converted to CT because conversion can be performed only by thyroid C cells. In other words, PCT that cannot be converted to CT may be increased during bacterial infections. As a result, we observed no significant correlation between PCT and CT in the infection group, with the correlation coefficient being similar in both groups in our study. In cases that showed higher PCT levels in the infection group, it may be believed that there would be a significant difference in the correlation coefficient between the 2 groups.

If PCT level is affected by HD, its measurement will not provide an accurate diagnosis and prognostic assessment of bacterial infection. Therefore, we investigated the effect of HD on PCT levels. In this study, PCT levels apparently decreased after HD using high-flux membranes, indicating that they are influenced by HD using high-flux membranes [23]. However, because low-flux membranes were not used in our study, we could not determine the effect of HD using low-flux membranes on PCT levels. When patients on HD using high-flux membranes develop bacterial infections, PCT levels immediately before HD should be investigated. In addition, in experimental sepsis, mortality is decreased by antiserum to PCT [24]. Therefore, elimination of PCT by HD using high-flux membranes may decrease mortality in patients with bacterial infections.

We investigated the use of PCT as a diagnostic marker of infection only in patients on HD. However, PCT has recently been identified as a marker of inflammation and has been recommended as a potential new marker of peritonitis in patients on continuous ambulatory peritoneal dialysis (CAPD) [25, 26]. CAPD patients also show increased CT and PCT synthesis in thyroid C cells because of chronic kidney disease. The results of these investigations on CAPD patients are concurrent with the results of the present study.

## 5. Conclusion

PCT was a very good marker of bacterial infection even in patients with increased CT levels undergoing HD. PCT levels should be determined before HD since the levels can be affected by high-flux membranes. Furthermore, the PCT cut-off level that indicates a bacterial infection should be set at 0.5 ng/mL in HD patients.

## Figures and Tables

**Table 1:** The comparing demographics of the two groups.

	Infection group (*N* = 15)	Noninfection group (*N* = 61)	*P* value
Age	65.1 ± 14.9	65.7 ± 12.6	N.S
Gender	(M: 10, F: 5)	(M: 32, F: 29)	N.S

*Localized infection*			
Pneumonia	13	0	—
Arteriovenous graft infection	1	0	—
*Systemic infection*			
No identifiable source	1	0	—

M: male; F: female. The data are expressed as the mean ± SD.

**Table 2:** Correlation between PCT and CT levels in the 2 groups.

	Infection group (*N* = 15)	Noninfection group (*N* = 61)
PCT (ng/mL)	3.8 ± 10.1	0.2 ± 0.14
CT (pg/mL)	78.7 ± 41.2	49.1 ± 23.8
*rs*	0.38	0.36
*p*	NS	0.005

PCT: procalcitonin; CT: calcitonin. The data are expressed as the mean ± SD.
